# Impact of Chronic Exposure to Arsenate through Drinking Water on the Intestinal Barrier

**DOI:** 10.1021/acs.chemrestox.3c00201

**Published:** 2023-10-11

**Authors:** Adrián Domene, Helena Orozco, Pilar Rodríguez-Viso, Vicente Monedero, Manuel Zúñiga, Dinoraz Vélez, Vicenta Devesa

**Affiliations:** Instituto de Agroquímica y Tecnología de Alimentos, Calle Agustín Escardino 7, 46980 Paterna, Spain

## Abstract

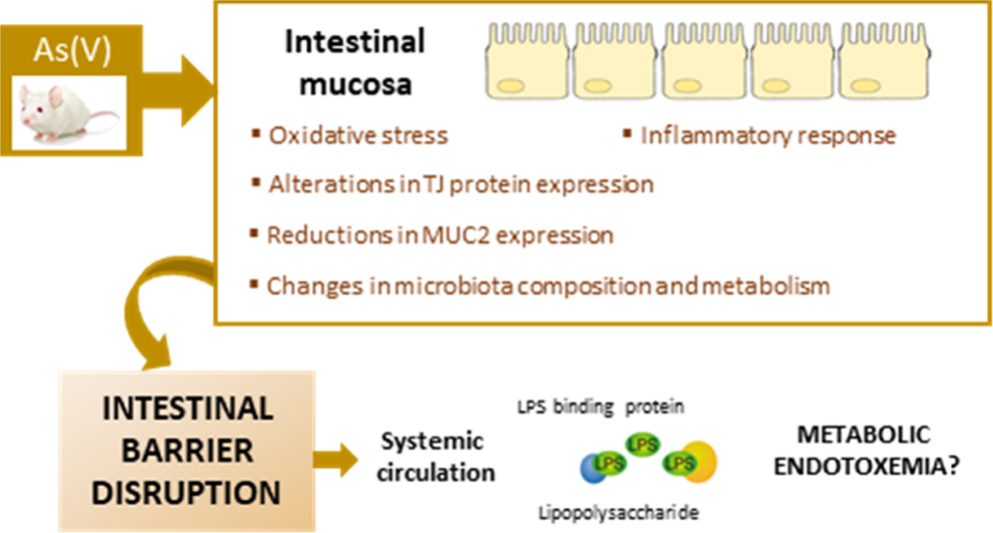

Chronic exposure to inorganic arsenic (As) [As(III) + As(V)], which affects millions of people, increases the incidence of some kinds of cancer and other noncarcinogenic pathologies. Although the oral pathway is the main source of exposure, *in vivo* studies conducted to verify the intestinal toxicity of this metalloid are scarce and are mainly focused on evaluating the toxicity of As(III). The aim of this study was to evaluate the effect of chronic exposure (6 months) of BALB/c mice to As(V) (15–60 mg/L) via drinking water on the different components of the intestinal barrier and to determine the possible mechanisms involved. The results show that chronic exposure to As(V) generates a situation of oxidative stress (increased lipid peroxidation and reactive species) and inflammation (increased contents of several proinflammatory cytokines and neutrophil infiltrations) in the intestinal tissues. There is also evidence of an altered expression of constituent proteins of the intercellular junctions (*Cldn1*, *Cldn3*, and *Ocln*) and the mucus layer (*Muc2*) and changes in the composition of the gut microbiota and the metabolism of short-chain fatty acids. All of these toxic effects eventually may lead to the disruption of the intestinal barrier, which shows an increased paracellular permeability. Moreover, signs of endotoxemia are observed in the serum of As(V)-treated animals (increases in lipopolysaccharide-binding protein LBP and the proinflammatory cytokine IL-1β). The data obtained suggest that chronic exposure to As(V) via drinking water affects the intestinal environment.

## 1 Introduction

Inorganic arsenic (As) can be found in nature mainly in two oxidation states: arsenate [As(V)] and arsenite [As(III)]. Both species are listed as carcinogenic to humans.^1^ However, the observed *in vitro* effects show that the trivalent form is more toxic.^2,3^ The greater toxicity of As(III) is linked to a higher cellular uptake and accumulation and a larger interaction with molecules essential for the maintenance of homeostasis.^2,4^

The routes of exposure to inorganic As vary depending on whether chronic hydroarsenicism is endemic in the region. In nonendemic areas, food, mainly cereals,^5^ are considered to be the main dietary sources. In these areas, the recommended intake, 3.0 μg As/kg body weight (bw) per day,^6^ is rarely exceeded. However, the European Food Safety Authority (EFSA), in its latest report on inorganic As, points to high consumers of rice- and algae-based food, as well as children under 3 years of age, as populations with remarkable intakes that can exceed those recommended.^5^ In endemic areas, the population is mainly exposed through the consumption of contaminated drinking water. These populations present high As intakes and As concentrations in biomarkers of exposure that exceed the toxicological reference values for all age groups.^7−9^

The assessment of As intakes by the chronically exposed populations does not include speciation of the inorganic form. Therefore, the ingested amounts of each inorganic species are unknown, a fact that is important from an epidemiological perspective since, as previously mentioned, each species has a different degree of toxicity. Speciation studies of As in various dietary sources show that As(V) generally predominates in drinking water,^10^ whereas the proportions of inorganic As species are more variable in foodstuffs. In rice, higher amounts of As(III) are reported,^11−13^ whereas in seafood products and seaweed, in general, As(V) is the predominant inorganic species.^14,15^ On the other hand, it has recently been reported that culinary processing can interconvert the chemical forms of inorganic arsenic.^16^ Thus, the population may be exposed to both inorganic species through the diet.

However, due to the higher toxicity of As(III) observed *in vitro*, toxicological studies have mainly focused on this arsenical form, including those studies aimed at investigating the inorganic As intestinal effects. Subchronic and chronic exposure to As(III) produces deleterious effects on both microbiota and various components of the intestinal mucosa in animal models,^17−19^ compromising the integrity of the intestinal barrier.^17,18^ Research on intestinal toxicity of As(V) in experimental animals is limited to a study conducted by Wang, Zhu, Li, Zheng, Ding, O’Connor, Zhu, and Xue,^20^ which revealed that exposure to the inorganic pentavalent form disturbs the gut microbiota of earthworms. *In vitro* studies using intestinal epithelial cell models indicate that both forms of inorganic As generate a pro-oxidant and proinflammatory response and that both can affect paracellular permeability,^21,22^ although As(III) toxicity is evidenced at lower concentrations, which are more relevant from an environmental point of view. However, this scenario possibly varies *in vivo* as it has been shown that As(V) metabolism can generate As(III) and other toxic methylated metabolites in animals and humans.^23^

Therefore, additional *in vivo* studies are required to determine the intestinal toxicity of As(V), which is possibly the major inorganic form of As in our diet. The present study aimed to assess the effect of chronic exposure to As(V) through drinking water on the different components that constitute the intestinal barrier and to determine the possible mechanisms involved in intestinal toxicity using BALB/c mice.

## 2 Materials and Methods

### 2.1 Animals and As(V) Treatments

Six-week-old female BALB/c mice weighing from 15 to 20 g (*n* = 36) were obtained from Envigo. They were kept under controlled conditions (12 h light/dark periods, 22 °C, and 75% humidity) at the animal production and experimentation facility of the University of Valencia. They were fed *ad libitum* with standard rodent chow with low inorganic As contents (2 ng/g). The experimental procedures were designed in accordance with the European Union Directive 2010/63/EU, presented according to the ARRIVE guidelines for reporting animal research and approved by the Agriculture, Fisheries, and Food Council of the Generalitat Valenciana (Spain).

Mice were randomly divided into four groups of nine animals. The first group served as the control, and to the other three groups, As(V) (Merck) was administered through drinking water at 15, 30, and 60 mg/L, equivalent to approximately 2.25, 4.5, and 9 mg/kg bw/day, for a period of 6 months. These doses were selected according to the toxicological data reported for mice chronically exposed to As(V).^24^ Drinking water was changed every 3 to 4 days with freshly prepared As(V) solutions. Animals′ weights, water and food consumption, physical appearance, behavior, and occult blood in feces (Hemoccult kit, Beckman Coulter) were monitored throughout the experiment. At the end of the exposure, mice were euthanized by inhalation of isoflurane and cervical dislocation. The intestine was immediately recovered, washed abundantly with 0.9% NaCl (m/v), separated into various fragments, and stored at −80 °C for subsequent analysis. Blood samples were collected in tubes, which were left to stand at room temperature for 60 min to allow clotting. Subsequently, they were centrifuged (2000*g*, 10 min), and serum was recovered.

### 2.2 Oxidative Stress in the Intestinal Tissues

The oxidative stress caused by As(V) at the intestinal level was evaluated by the determination of the contents of reactive oxygen or/and nitrogen species (ROS/RNS), the level of lipid peroxidation, and the concentrations of reduced glutathione (GSH) in tissue from the small and large intestine.

The determination of ROS/RNS was assessed by the 2′,7′-dichlorodihydrofluorescein diacetate (H_2_DCF) assay. Intestinal tissues were homogenized in modified radioimmunoprecipitation assay (RIPA) buffer [NaCl 150 mM (Panreac), Tris-HCl 50 mM pH 8 (Sigma), EDTA 5 mM (Sigma), Triton X-100 1% v/v (Merck), sodium deoxycholate 0.5% m/v (Sigma), and protease inhibitor mix (Roche) 6 mg/mL] at a proportion of 1:20 using a tissue disruptor (Qiagen). After centrifugation (8000*g*, 5 min), the supernatants (100 μL) were incubated with 6 μL of 10 μM H_2_DCF (Sigma) for 30 min. The fluorescence of the mixture was analyzed at excitation/emission wavelengths of 488/530 nm in a CLARIOstar microplate reader (BMG-Labtech).

Lipid peroxidation was determined using a thiobarbituric acid reactive substance assay (TBARS). Tissue homogenates obtained as described previously (20 μL) were incubated with 20 μL of 8.1% (m/v) sodium dodecylsulfate (SDS, GE healthcare), 150 μL of acetic acid 20% v/v (Janssen), 150 μL of thiobarbituric acid 0.8% v/v (TBA, Sigma), and 60 μL of distilled H_2_O in a water bath at 95 °C for 60 min. After cooling, the mixture was extracted with 200 μL of 1-butanol (Panreac), and the organic phase obtained after centrifugation (1300*g*, 4 min) was analyzed using the CLARIOstar microplate reader (λ excitation = 532; λ emission = 553).

For GSH determination, intestinal tissues (10 mg) were homogenized with 400 μL of ammonium phosphate buffer 0.1 M with EDTA 10 mM pH 8 (Sigma) and 100 μL of orthophosphoric acid 25% v/v (Fluka) at a proportion of 1:50. After centrifugation (8000*g*, 20 min, 4 °C), 10 μL of the supernatant was incubated with 10 μL of de *o*-phthalaldehyde 0.07 M (Fluka) and 180 μL of phosphate buffer 0.1 M-EDTA 10 mM pH 8. The mixture was analyzed using the CLARIOstar microplate reader (λ excitation = 340 nm; λ emission = 420 nm).

### 2.3 Proinflammatory Response in the Intestinal Tissues

To determine the proinflammatory response produced by As(V) exposure, the intestinal tissue contents of several proinflammatory cytokines/chemokines and the neutrophil infiltration of the mucosa were analyzed.

#### 2.3.1 Evaluation of Tissue Contents of Proinflammatory Cytokines/Chemokines

The tissue of the small and large intestine (50 mg) was homogenized in a modified RIPA buffer in a 1:20 proportion. After centrifugation (8000*g*, 5 min, 4 °C), tissue homogenates were used to determine the cytokines by means of the following specific ELISA kits: IL-6 (Invitrogen), IL-1β (Invitrogen), TNF-α (Invitrogen), and IL-17A (MyBioSource).

#### 2.3.2 Evaluation of the Neutrophil Infiltration

The infiltration of neutrophils was assessed by histological examination (Section 2.5.1) and by the analysis of lactoferrin in fresh feces of the control and As(V)-treated animals collected at the end of the experiment. For lactoferrin analysis, feces (50 mg) were homogenized at a 1:10 proportion in modified RIPA buffer with a bead beater (FastPrep-24 5G Instrument, MP Biomedical), using 0.1 mm glass beads and three cycles of 40 s at a 6 m/s speed, with 1 min intervals in which samples were kept on ice. The fecal contents of lactoferrin in the homogenates were analyzed using a specific ELISA kit (MyBioSource).

#### 2.3.3 Signaling Pathways Activated by As(V) Exposure

The identification of possible pathways involved in As(V) toxicity was performed by Western blotting. Samples of large intestine were homogenized in modified RIPA buffer at a proportion of 1:20, and protein content was measured with a Nanodrop ND-1000 (NanoDrop Technologies). Homogenates (30 μg protein/lane) were loaded in a 10% acrylamide gel in an electrophoresis cuvette with running buffer (Tris 25 mM pH 8.3, glycine 192 mM, SDS 0.1% m/v). Electrophoresis was carried out at 80 V for 30 min, followed by 120 V for 1 h. Afterward, proteins were transferred to poly(vinylidene fluoride) (PVDF) membranes (Immobilon) in a semidry protein transfer system (Bio-Rad) for 1 h at 100 mA. Following the transfer, membranes were blocked with a solution of 5% (m/v) albumin (Sigma) in Tris-buffered saline containing 0.1% (v/v) Tween-20 (TBST) for 1 h at room temperature and incubated overnight at 4 °C with primary antibodies according to the manufacturer’s recommendations. Primary antibodies were as follows: antiphospho-p38 MAPK (D3F9, 1:1000, Cell Signaling Technology), antiphospho-SAPK/JNK (81E11, 1:1000, Cell Signaling Technology), and antiphospho-IKK α/β (2697, 1:1000, Cell Signaling Technology). After being washed in TBST, membranes were incubated for 1 h with a horseradish peroxidase-conjugated goat antirabbit antibody (1:10 000, Amersham) diluted in a blocking buffer. Membranes were subsequently washed, incubated with the Amersham ECL Select Western Blotting Detection Reagent (GE Healthcare), and exposed with an ImageQuant 800 apparatus (Cytiva). All measurements were normalized by hybridization with the housekeeping β-actin (1:10 000, Sigma) or HPRT1 (1:10 000, Cohesion Bioscience) antibodies. The bands were quantified by using the densitometry program ImageJ V1.8.0.

### 2.4 Study of the Intestinal Microbiota and Its Metabolism

#### 2.4.1 Composition of the Microbiota

Total DNA was extracted from mice fecal pellets obtained at the end of the experimental period using the QIAamp DNA stool Mini Kit (Qiagen) following the instructions of the manufacturer. Isolated DNA was quantified with a Qubit 2.0 fluorometer (Invitrogen). Variable V3 and V4 regions of the 16S rDNA gene were amplified following the 16S rDNA gene Metagenomic Sequencing Library Preparation Illumina protocol (Cod. 15044223 Rev. A). Gene-specific primers (PCR1_f: 5′-TCGTCGGCAGCGTCAGATGTGTATAAGAGACAGCCTACGGGNGGCWGCAG-3′; PCR1_r: 5′-GTCTCGTGGGCTCGGAGATGTGTATAAGAGACAGGACTACHVGGGTATCTAATCC-3′) containing Illumina adapter overhang nucleotide sequences were selected according to Klindworth, Pruesse, Schweer, Peplies, Quast, Horn, and Glöckner.^25^ After 16S rDNA gene amplification, the multiplexing step was performed using a Nextera XT Index Kit. We run 1 μL of the PCR product on a Bioanalyzer DNA 1000 chip to verify the amplicon size (∼550 bp) on a Bioanalyzer (Agilent). After size verification, the libraries were sequenced using a 2  ×  300 pb paired-end run (MiSeq Reagent kit v3) on a MiSeq Sequencer according to the manufacturer’s instructions (Illumina). DNA amplification and sequencing were carried out at the Genomics Unit of the Central Service for Experimental Research of the University of Valencia.

All subsequent analyses were performed in R version 4.1.1. 16S–V4 raw forward and reverse reads were processed with the DADA2 package.^26^ Reads were trimmed to 260 bases and filtered with maxEE set to 5. Reads were subsequently dereplicated, errors were estimated, and sequences merged using functions implemented in the DADA2 package. Chimeric sequences were removed using the function removeBimeraDenovo from the DADA2 package with the method set to consensus. Taxonomy was assigned to amplicon sequence variants (ASVs) using SILVA version 138.^27^ Initial preprocessing and data exploration were performed using functions implemented in the R package phyloseq version 1.38.0.^28^ Graphic outputs were obtained with the ggplot2 package.^29^ ASVs not assigned to any phylum, as well as ASVs with 10 or fewer reads, were removed from the data set. Potential contaminant sequences were identified by using the decontam package,^30^ with the method set to frequency and threshold 0.3. For phylogenetic reconstruction, ASV sequences were aligned with tools implemented in the DECIPHER package version 2.22.0^31^ and phylogeny was inferred using a GTR + G + I (generalized time-reversible with γ rate variation and invariants) maximum likelihood phylogenetic tree with the phangorn package version 2.7.1.^32^

Alpha diversity was analyzed by using the breakaway package version 4.7.6^33^ for richness and the DivNet package version 0.4.0^34^ for diversity. For beta diversity analysis, zero counts were replaced by imputed values using the square root Bayesian multiplicative method implemented in zCompositions version 1.4.^35^ Euclidian distances were calculated (dist, base R 4.1.1) by first carrying out the phylogenetic isometric log-ratio transformation as implemented in the philr package.^36^ Calculated distances were used to obtain PCA or NMDS coordinations using the tools implemented in the phyloseq package. Significant differences between treatment groups were estimated by pairwise PERMANOVA calculations using the adonis.pair function included in the package EcolUtils (https://github.com/GuillemSalazar/EcolUtils) that utilizes the adonis function of the VEGAN package.^37^ Homogeneity of variances was checked by using the functions betadisper and permutest implemented in the VEGAN package. Differential abundances of taxa between treatment groups were estimated by two approaches: beta-binomial count regression models from the R package corncob^38^ that account for the within-sample taxa correlation and variable sequencing depth, and analysis of the composition of microbiomes with bias correction (ANCOM-BC) modeling, which estimates a change between test groups for each taxon using log-transformed values of absolute sequence counts.^39^

#### 2.4.2 Fecal Contents of Short-Chain Fatty Acids (SCFA)

Feces (50 mg) were homogenized in 1 mL of isobutanol 10% (Fluka) with 0.1 mm glass beads, as described in Section 2.3.2. The homogenate (675 μL) was mixed with 125 μL of NaOH 20 mM (Panreac) and 23 μL of hexadecanoic acid 50 mg/L (Merck) as the internal standard. Afterward, a portion of the mixture (400 μL) was derivatized with 80 μL of isobutanol, 100 μL of pyridine (Sigma), and 50 μL of isobutyl chloroformate (Sigma). SCFA-derived esters were extracted with 150 μL of *n*-hexane (Supelco), and the organic phase was analyzed by gas chromatography coupled to mass spectrometry (GC-MS, Agilent 5977B GC/MSD).

SFCA esters were separated in a DB-5 MS Ultra Inert capillary column (30 m × 0.25 mm id, 0.25 μm) (Agilent) in split mode (1:50) and using helium as the carrier gas (1 mL/min). The temperature of the GC-MS ion source and the transfer line was set at 250 °C. The oven temperature gradient was as follows: 5 min at 40 °C, 4 °C/min increase until 90 °C. and an increase of 30 °C/min until 290 °C. The compounds were identified from whole scanning data (*m*/*z* 35–500) using the NIST 17 library of mass spectra (2011 version, Agilent). Data were processed using Agilent MassHunter Qualitative Analysis (version B.06.00) software, and the quantification was performed using standard solutions of acetic acid (Janssen Chimica), propionic acid (Fluka), isobutyric acid (Sigma), and butyric acid (Fluka). Standards solutions were derivatized under the same conditions as those applied to feces samples. The analytical features of the method were reported previously.^18^

### 2.5 Evaluation of the Intestinal Mucosa Structure

The structural evaluation of the mucosa was carried out by histological examination and analysis of the expression of proteins of the mucus layer and the intercellular junctions.

#### 2.5.1 Histological Evaluation

Tissues (approximately 1 cm) were immersed in a solution of paraformaldehyde 4% in PBS (PAF, Sigma) immediately after the sacrifice and stored at 4 °C until the moment of their preparation. Afterward, the intestinal portions were embedded in paraffin (Merck) and sectioned at 5 μm thickness on a microtome (Leica Biosystems). The sections were first deparaffinized and then stained with hematoxylin and eosin (H&E, Abcam) to assess the degree of neutrophil infiltration (Section 2.3.2).

For goblet cell examination, periodic acid-Schiff staining was used. Deparaffinized and rehydrated sections were treated with a periodic acid (Panreac) for 5 min. Slides were washed with distilled water and stained with Schiff’s reagent (Sigma) for 15 min. Finally, the sections were counterstained with hematoxylin. Images were acquired by using a Nikon Eclipse 90i epifluorescence microscope.

#### 2.5.2 Analysis of the Expression of Proteins of the Tight Junctions and the Mucus Layer

The gene expression of mucin (*Muc2)* and the tight junctions’ proteins [claudin 1 (*Cldn1*), claudin 3 (*Cldn3*), and occludin (*Ocln*)] was evaluated by RT-qPCR. Large intestinal portions (∼0.5–1 cm) were transferred to RNA later (Qiagen) immediately after sacrifice and kept for 24 h at 4 °C. After the removal of RNA, later samples were stored at −80 °C until analysis. RNA extraction was performed with a NucleoSpin RNA II kit (Macherey-Nagel). Total RNA was quantified with a Nanodrop ND-1000, and quality was checked by agarose electrophoresis. First-strand complementary DNA (cDNA) was obtained from 200 ng of total RNA using the Reverse Transcriptase Core kit (Eurogentec Headquarters) according to the instructions of the manufacturer. qPCR was performed using the LightCycler 480 Real-Time PCR system (Roche Diagnostics). Reactions were carried out in a final volume of 10 μL containing 5 μL of LightCycler 480 SYBR Green I Master Mix (2×, Roche), 2.5 μL of cDNA (20 ng/μL), and 1 μL of each forward and reverse primer (10 μM; Biolegio). The qPCR program consisted of an initial incubation at 95 °C for 5 min, followed by 40 cycles: 10 s denaturation at 95 °C, 10 s annealing at 55 °C, and 20 s elongation at 72 °C. The sequence and efficiency of the primers used in the study are detailed in Table 1. Data were normalized using reference genes *Rn18S* and *Hprt1* (Table 1) and analyzed with REST-384 software.^40^

**Table 1:** Sequence and Efficiency of the Oligonucleotides Used in This Study

Gen	GenBank ID	sequence 5′-3′	amplicon (bp)	efficiency
*Rn18S*	NM_023566.3	F: CGGACAGGATTGACAGATTG	98	1.83 ± 0.10
		R: CAAATCGCTCCACCAACTAA		
*Hprt1*	NM_013556.2	F: GTTAAGCAGTACAGCCCCAAA	131	1.98 ± 0.06
		R: AGGGCATATCCAACAACAAACTT		
*Muc2*	NM_023566.4	F: GCTGACGAGTGGTTGGTGAATG	135	2.22 ± 0.01
		R: GATGAGGTGGCAGACAGGAGAC		
*Cldn1*	NM_016674.4	F: AGGTCCTGGCGACATTAGTGG	204	2.05 ± 0.03
		R: CGTGGTGTTGGGTAAGAGGT		
*Cldn3*	NM_009902.4	F: TCATCGGCAGCAGCATCATCAC	256	1.74 ± 0.02
		R: ACGATGGTGATCTTGGCCTTGG		
*Ocln*	NM_008756.2	F: ATGTCCGGCCGATGCTCTC	308	1.97 ± 0.18
		R: TTTGGCTGCTCTTGGGTCTGTAT		

### 2.6 Evaluation of the Intestinal Barrier Integrity and Biomarkers of Endotoxemia

Intestinal permeability was evaluated by determining the concentration of albumin in feces. Feces (∼50 mg) were homogenized in 0.5 mL of modified RIPA buffer using a bead beater, as described in Section 2.3.2. The homogenate was centrifuged (8000*g*, 5 min, 4 °C), and the concentration of fecal albumin was determined in the supernatant using the Mouse Albumin ELISA kit (Cusabio), following the manufacturer’s instructions.

To assess possible signs of endotoxemia, proinflammatory cytokine IL-1β and lipopolysaccharide-binding protein (LBP) in serum were assessed using specific ELISA kits [LBP (Cloud-Clone Corporation), IL-1β (Sigma)].

### 2.7 Statistical Analysis

The *t*-student test or one-way analysis of variance (ANOVA) with multiple *post hoc* comparisons (Fisher HSD test) was employed when the requirements for normality (Shapiro–Wilk test) and homogeneity of variances between all groups (Brown–Forsythe test) were met. For the rest of the cases, data were analyzed with the Mann–Whitney U-test or the Kruskal–Wallis test with multiple comparisons using the Dunn test. Differences were considered statistically significant at *p* < 0.05. The analyses were performed with SigmaPlot 14.5 (Systat Software Inc.). The correlation heatmap was performed in R software (version 4.1.1) using various packages: Hmisc, ggplot2, and purr.

## 3 Results

### 3.1 Health Status of Animals during As(V) Exposure

Chronic exposure to As(V) did not affect the general health status of the mice, and no changes in behavior were observed. Food and water consumption was comparable to those observed for the control group, and no appreciable changes in stool frequency were detected. However, there was an increase of fecal occult blood in As(V) treatments (Table S1, Supporting Information). At week 19, no occult blood was observed in the feces of the control group; however, As(V)-treated animals displayed 15 positives (15 mg/L: 4, 30 mg/L: 4, 60 mg/L: 7). At week 22, only one animal showed fecal occult blood in the control group, while 4, 6, and 7 animals were positive in the 15, 30, and 60 mg/L groups, respectively. These data constitute the first indication of a possible adverse effect of As(V) at the intestinal level.

### 3.2 Redox Status of Intestinal Tissues

**Figure 1:**
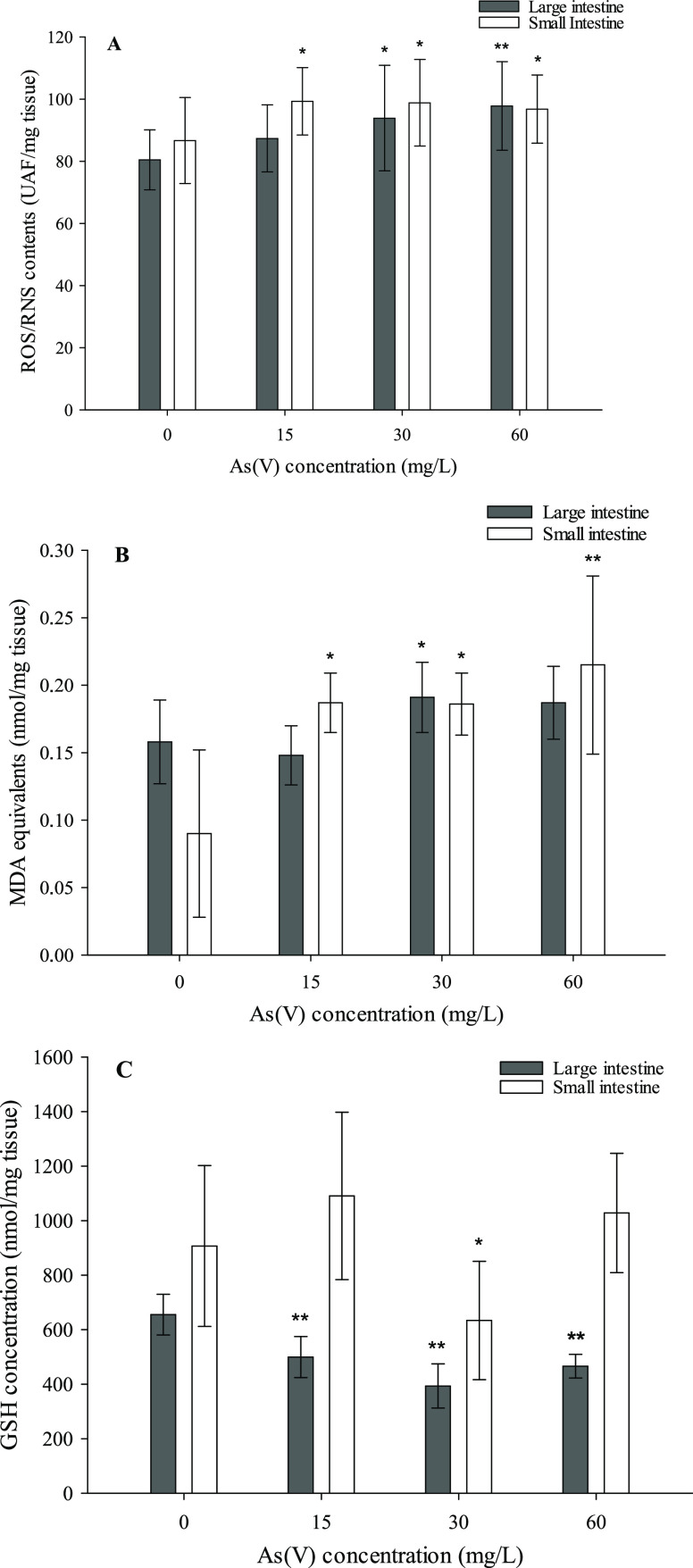
Oxidative stress markers in control animals and animals treated with As(V). (A) ROS/RNS levels analyzed by the H2DCF technique [AUF (arbitrary units of fluorescence)/mg tissue]. (B) Lipid peroxidation estimated as MDA (malondialdehyde) equivalents (nmol/mg tissue). (C) GSH concentration (nmol/mg tissue). Values are expressed as the mean ± SD (*n* = 9). Asterisks indicate statistically significant differences with respect to nontreated animals (**, *p* < 0.001; *, 0.001 ≤ *p* < 0.05).

Figure 1A shows data on H_2_DCF oxidation; in all As(V) treatments, a statistically significant increase of ROS/RNS levels in the small intestine was evidenced (15 mg/L, 14%; 30 mg/L, 14%; 60 mg/L, 11%). In the large intestine, however, this increase was only significant at the highest concentrations (30 mg/L, 17%; 60 mg/L, 21%).

Lipid peroxidation data also confirmed intestinal oxidative stress caused by As(V) (Figure 1B). Compared with nontreated mice, an increase in lipid peroxides was observed in As(V)-exposed animals, the changes in the small intestine (15 mg/L, 110%; 30 mg/L, 108%; 60 mg/L, 147%) being greater than those observed in the large intestine (30 mg/L, 21%). In addition, a decrease in tissue GSH was evidenced as a consequence of chronic exposure to As(V) (Figure 1C), confirming this pro-oxidant response. GSH was reduced in both portions of the intestine, being greater in the large intestine (15 mg/L, 24%; 30 mg/L, 40%; 60 mg/L, 29%) than in the small intestine (30 mg/L, 31%).

### 3.3 Inflammatory Status of the Intestinal Tissues

#### 3.3.1 Intestinal Proinflammatory Cytokines Levels

**Figure 2:**
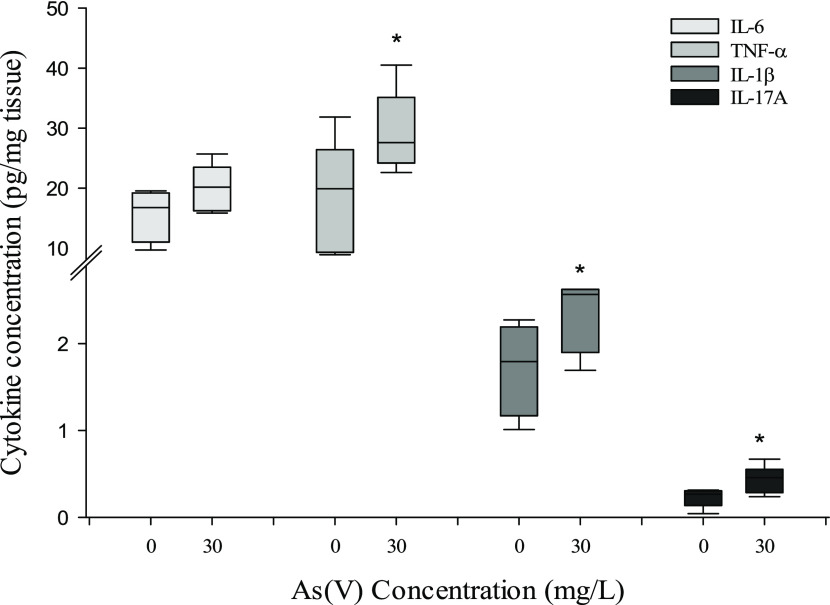
Concentration of proinflammatory cytokines (IL-6, TNF-α, IL-1β, and IL-17A) in distal colon samples of the control and animals treated with 30 mg/L As(V). Values expressed as pg/mg tissue (mean ± SD, *n* = 9). Asterisks indicate statistically significant differences with respect to untreated animals (*p* < 0.05).

The results obtained for the large intestine showed no change in cytokine levels among treated and untreated animals in one of the fragments tested, the most proximally located (data not shown); however, significant differences were evidenced in a more distal section of the colon (Figure 2). Distal colon fragments were obtained only from control animals and those exposed to 30 mg/L. The cytokine with the highest increase was IL-17A (99%), followed by TNF-α (54%) and IL-1β (46%). The variations observed for IL-6 (increase over control: 30%) were not statistically significant.

#### 3.3.2 Neutrophil Infiltration Markers

**Figure 3:**
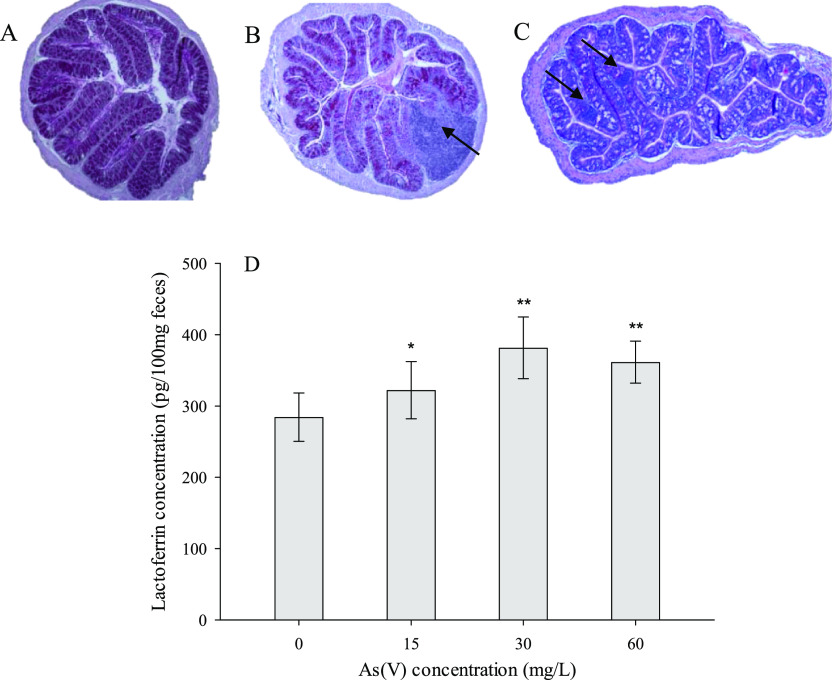
PAS-stained cross sections of the colon from a control animal (A) and an animal treated with 15 mg/L (B) or 30 mg/L (C) of As(V). Black arrows point to lymphocyte infiltration. Magnification ×4. Lactoferrin contents (D) expressed as pg/100 mg feces (mean ±. SD, *n* = 9). Asterisks indicate statistically significant differences with respect to nontreated animals (**, *p* < 0.001; *, 0.001 ≤ *p* < 0.05).

Figure 3A shows sections of the colon of control animals (3A) and animals treated with 15 mg/L (3B) and 30 mg/L (3C) of As(V). All treatments showed infiltrates, although these were more frequent in As(V)-treated animals. Of the 9 animals tested in the control group, only 3 showed infiltrates in the colonic sections. In the groups treated with the highest concentrations (30 and 60 mg/L), all of the animals (*n* = 9) showed infiltrates, while at 15 mg/L, 4 out of the 5 animals tested showed infiltrates. Fecal lactoferrin data support this infiltration process (Figure 3D). The fecal lactoferrin contents found in treated mice were higher compared to untreated mice (15 mg/L, 13%; 30 mg/L, 34%; 60 mg/L, 27%), suggesting the induction instead of generation of a proinflammatory process.

#### 3.3.3 Activation of Pro-oxidant/Proinflammatory Signaling Pathways

**Figure 4:**
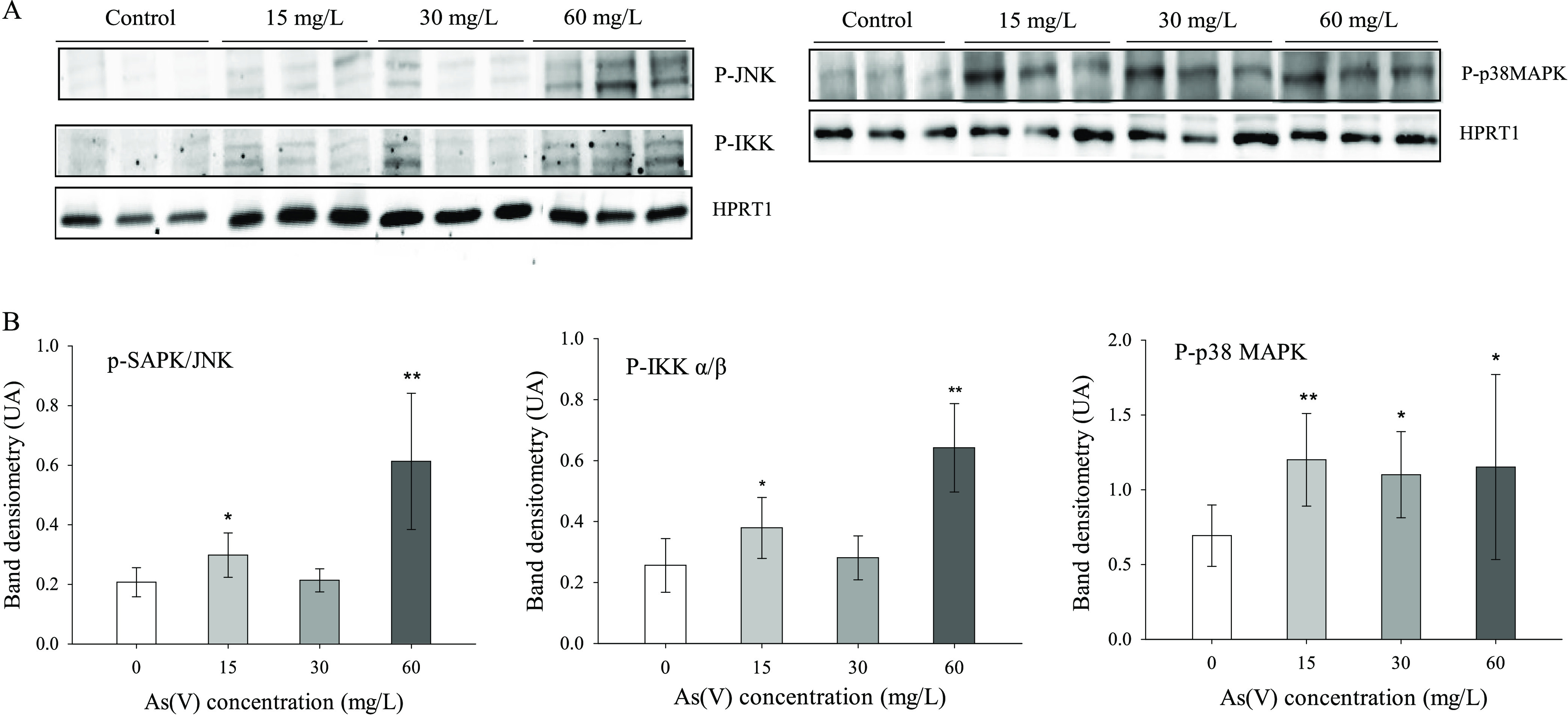
Activation of signaling pathways in the colon by As(V) exposure. (A) Western blot of phospho-p38, phospho-SAPK/JNK, and phospho-IKKα/β. Only three representative samples from each treatment are shown. The loading control was HPRT1. (B) Levels of phosphorylated forms quantified by densitometry and expressed as arbitrary units (AU, mean ± SD, *n* = 6). Asterisks indicate statistically significant increases with respect to nontreated animals (**, *p* < 0.001; *, 0.001 ≤ *p* < 0.05).

Figure 4 shows the levels of phosphorylated forms of p38, IKK, and JNK in the colon of the control and As(V)-treated animals, analyzed by Western blotting. The data point to the activation of p38 (percentage of increase with respect to the control animals: 59–73%), SAPK/JNK (44–196%), and NFκB (48–150%) pathways. The activation of specific signal transduction pathways involved in inflammatory processes is further evidence of a proinflammatory/pro-oxidant response at the intestinal level in As(V)-treated animals.

### 3.4 Alterations of Intestinal Microbiota and Its Metabolism in As(V)-Treated Animals

#### 3.4.1 Intestinal Microbiota Composition

**Figure 5:**
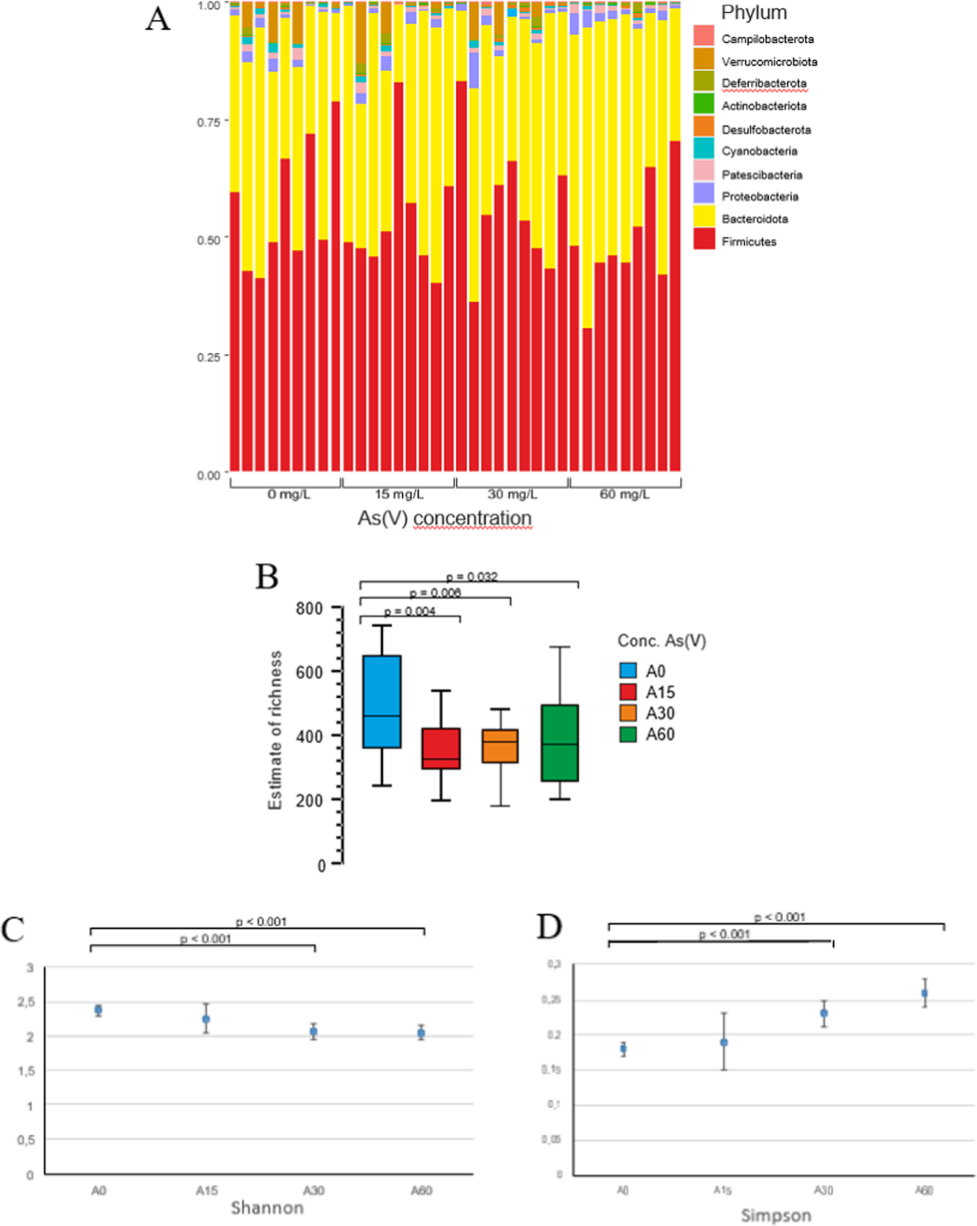
Analysis of microbiota composition in the control and As(V)-exposed mice. (A) Stacked bar plot showing the relative abundance of phyla in mice fecal samples; each bar represents an individual mouse fecal sample. (B) Estimates of taxonomic richness of mouse fecal samples. (C, D) Estimates of Shannon’s and Simpson’s indices, respectively. Brackets indicate groups compared with significant *p*-values (<0.05).

The effect of chronic exposure to As(V) on intestinal microbiota was estimated by analyzing fecal samples collected at the end of the experiment. At the phylum level, Firmicutes and Bacteroidetes were the most prevalent taxa (Figure 5A), as previously reported for laboratory mice.^41^ Analysis of alpha diversity showed significant decreases in species richness at all As(V) concentrations (Figure 5B). Furthermore, estimates of the indexes of Shannon and Simpson revealed significant differences between the control group and those exposed to 30 or 60 mg/L As(V) (Figure 5C,D), although no significant difference was detected for the group exposed to 15 mg/L. The trends in both indexes agreed with the estimated decreased richness. As both diversity indexes integrate richness and evenness, this result suggests that exposure to As(V) altered the relative abundance of some taxa.

Beta diversity estimations showed significant differences in the composition of the fecal microbiota between the control mice and those exposed to As(V). PCoA and NMDS ordinations did not show a clear clustering of samples according to As(V) exposure (data not shown). However, PERMANOVA analysis accounting for the cage as a confounding factor revealed significant differences among the treatment groups (*R*^2^ 0.186 and corrected P-value 0.001). In agreement with this, pairwise PERMANOVA analyses detected only no significant differences for the groups exposed to 15 or 30 mg/L As(V) (Table 2). The analysis of the homogeneity of variances did not detect significant differences between the four groups of samples, thus supporting the reliability of the PERMANOVA analyses (Figure S1).

**Table 2:** Results of Pairwise PERMANOVA Analyses of the Different Treatment Groups Used in This Study

combination	sum of squares	mean squares	*F* model	*R*^2^	*p*-value	*p* corrected
AV0 vs AV15^a^	0.062	0.062	2.325	0.127	0.026	0.031
AV0 vs AV30	0.066	0.066	2.683	0.144	0.008	0.018
AV0 vs AV60	0.111	0.111	4.977	0.237	0.001	0.006
AV15 vs AV30	0.031	0.031	1.117	0.065	0.359	0.359
AV15 vs AV60	0.052	0.052	2.053	0.114	0.026	0.031
AV30 vs AV60	0.043	0.043	1.867	0.104	0.009	0.018

a AV0: control group; AV15: group exposed to 15 mg/L As(V); AV30: group exposed to 30 mg/L As(V); AV60: group exposed to 60 mg/L As(V).

**Figure 6:**
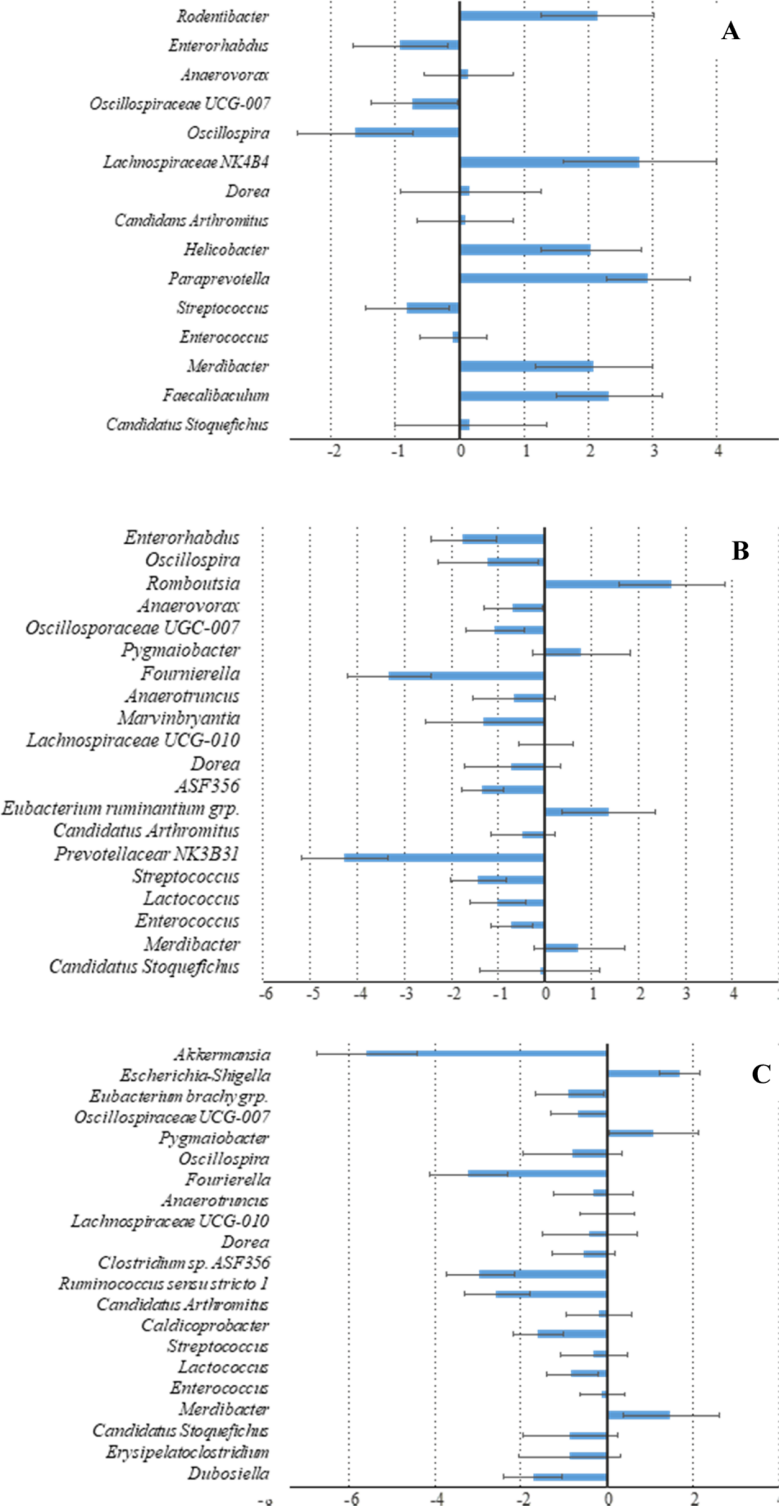
Differential abundance at the genus level represented by effect size (log fold change) derived from ANCOM-BC. Error bars represent standard errors. (A) Mice exposed to 15 mg/L As(V) against control mice. (B) 30 mg/L As(V). (C) 60 mg/L As(V).

Differential taxa were identified by using ANCOM-BC, excluding taxa with a proportion of zeros greater than 0.9 and considering structural zeros. The analysis detected 224 ASVs (amplicon sequence variants) with significant differences in samples treated with 15 mg/L As(V), 215 ASVs with 30 mg/L As(V), and 251 ASVs with 60 mg/L As(V) (results not shown). Reanalyzing the data at the family level, however, only 5 families with differences were detected with 15 mg/L As(V), 4 with 30 mg/L As(V), and 10 with 60 mg/L As(V). Only the *Enterococcaceae* family was detected in more than one treatment group. Analysis at the genus level detected 15 genera with differences with 15 mg/L As(V), 20 with 30 mg/L As(V), and 22 with 60 mg/L As(V) (Figure 8). At this level, 16 genera were detected in more than one treatment group. This result suggests that changes in taxa abundance, either direct or indirect, as a result of exposure to As(V) were specific to species or strains. Since accurate identification at the species level with our data set was limited to a relatively low number of ASVs, the results of the analysis at the genus level will be discussed. Most genera detected as differentially abundant belonged to the phylum Firmicutes. In contrast, among Bacteroidota, only the genus *Paraprevotella* was detected as significantly more abundant in mice treated with 15 mg/L As(V) and the genus *Prevotellaceae* NK3B31 as significantly less abundant with 30 mg/L As(V). On the whole, apparently, Bacteroidota were slightly affected by As(V). For some genera, a general trend was observed in all treatment groups. Genera *Oscillospira* and *Oscillospiraceae* UCG-007 were consistently detected as being significantly less abundant in the three groups exposed to As(V) (Figure 6). Genus *Enterococcus* and *Streptococcus* (order *Lactobacillales*) were also detected as significantly less abundant in the three groups, whereas the closely related genus *Lactococcus* was significantly less abundant in the groups treated with 30 or 60 mg/L As(V). The *Akkermansia*, *Fournierella*, and *Ruminococcus torques* group also showed a general trend to decreased abundance as As(V) concentration increased, although they were detected as significantly less abundant only in groups of animals exposed to 30 or 60 mg/L As(V) (Figures 8 and S2). In general, fewer genera were detected as significantly more abundant, except in the group exposed to 15 mg/L As(V) (Figures 6 and S2). Of note, the *Escherichia–Shigella* group displayed a trend of increased abundance, although it was only detected as significantly more abundant in the group exposed to 60 mg/L As(V) (Figures 6 and S2).

#### 3.4.2 SCFA Metabolism

**Figure 7:**
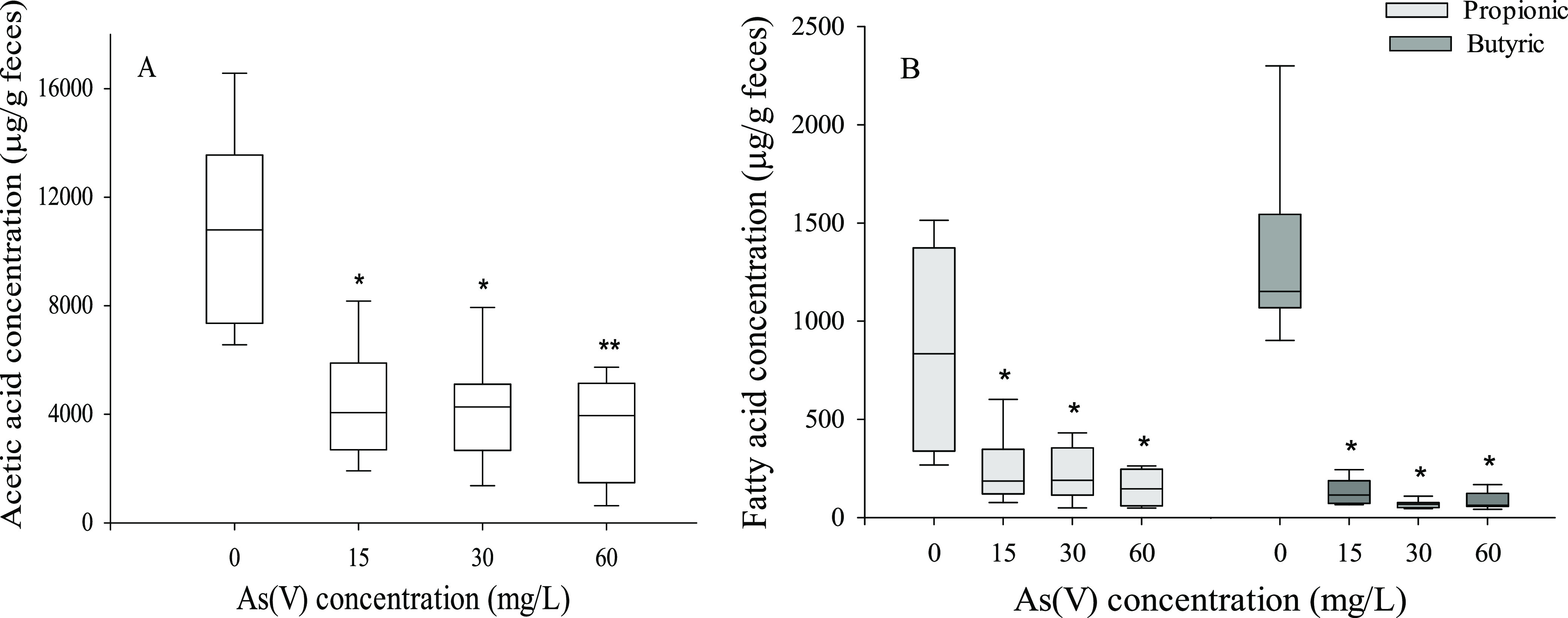
Fecal short-chain fatty acid concentrations (SCFA) of the control and As(V)-treated animals (A, acetic acid; B, propionic and butyric acid). Values expressed as μg of SCFA/g of feces (*n* = 9). Asterisks indicate statistically significant differences with respect to nontreated animals (**, *p* < 0.001; *, 0.001 ≤ *p* < 0.05).

**Figure 8:**
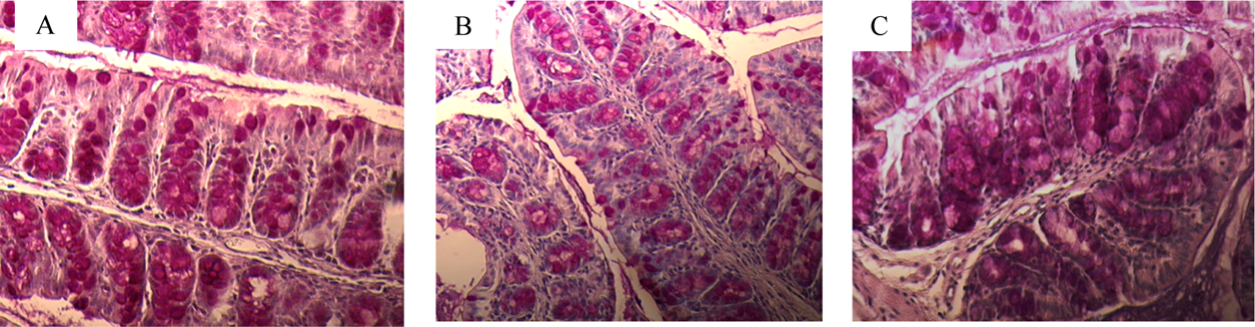
Effect of As(V) on the mucosecretory cells. PAS/hematoxylin-stained cross sections of the colon of a control animal (A), animals treated with 15 mg/L As(V) (B), and the ones treated with 30 mg/L As(V) (C). Magnification 16×.

Figure 7 shows the concentrations of SCFAs in the control and As(V)-treated animals. As(V) treatments reduced the luminal contents of SCFAs, butyric acid being the fatty acid with the greatest reductions (15 mg/L, 98%; 30 mg/L, 97%; 60 mg/L, 98%, Figure 8B), followed by propionic acid (15 mg/L, 78%; 30 mg/L, 78%; 60 mg/L, 86%, Figure 8B) and acetic acid (15 mg/L, 62%; 30 mg/L, 62%; 60 mg/L, 63%, Figure 8A).

### 3.5 As(V) Alters the Expression of Proteins of the Tight Junctions and the Mucus Layer

Table 3 shows the relative gene expression of the tight-junction proteins (*Cldn1, Cldn3, and Ocln*) and the mucin *Muc2* of the mucus layer in the colon of animals treated with As(V) with respect to the untreated animals. The treatment with As(V) generated a downregulation of *Muc2*, although a hormetic response was observed so that the downregulations were greater at the lowest concentrations of As(V) in drinking water. A similar trend was observed for the tight-junction proteins. Genes coding for Cldn1 and Ocln were statistically significantly downregulated in animals treated with 15 and 30 mg/L As(V), whereas all treatments with As(V) generated statistically significant downregulation of *Cldn3*.

**Table 3:** Relative Expression of *Muc2*, *Cldn1*, *Ocln*, and *Cldn3* in Mice Chronically Exposed to As(V) with Respect to Nontreated Animals^a^

gen	15 mg/L	30 mg/L	60 mg/L
*Muc2*	0.33 ± 0.10*	0.41 ± 0.11*	0.51 ± 0.18*
*Cldn1*	0.44 ± 0.13*	0.67 ± 0.14*	0.85 ± 0.33
*Ocln*	0.51 ± 0.16*	0.57 ± 0.15*	0.71 ± 0.28
*Cldn3*	0.62 ± 0.19*	0.68 ± 0.18*	0.26 ± 0.12*

a Values expressed as fold changes (mean ± SE, *n* = 9). An asterisk indicates statistically significant downregulation with respect to control animals (*p* < 0.05).

In addition, a statistically significant decrease in the amount of mucosecretory cells per crypt was observed in the colonic tissue of As(V)-treated mice with respect to nontreated animals [PAS+/crypt cells: control (16 ± 3); 15 mg/L (13 ± 1); 30 mg/L (13 ± 2)] (Figure 8). This could partly explain the lower expression of *Muc2* in the animals treated with the metalloid.

### 3.6 Effect of As(V) on the Intestinal Permeability and Biomarkers of Metabolic Endotoxemia

**Figure 9:**
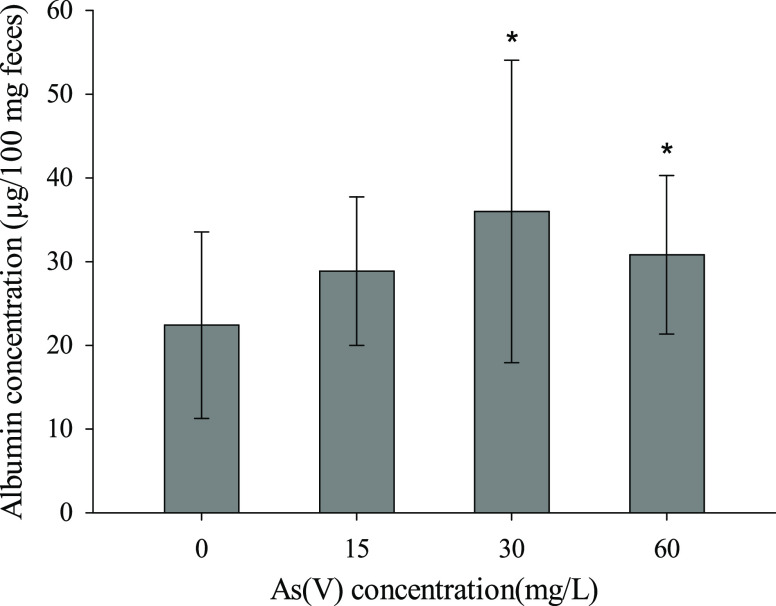
Fecal albumin contents (μg/100 mg of feces) in control animals and animals treated with As(V). Asterisks indicate statistically significant differences with respect to control animals (*p* < 0.05).

Fecal albumin quantification was used as a marker of intestinal permeability (Figure 9). The data showed significant increases in the As(V)-treated animals exposed to the highest concentrations compared to the nontreated mice (30 mg/L: 94%; 60 mg/L: 74%).

**Figure 10:**
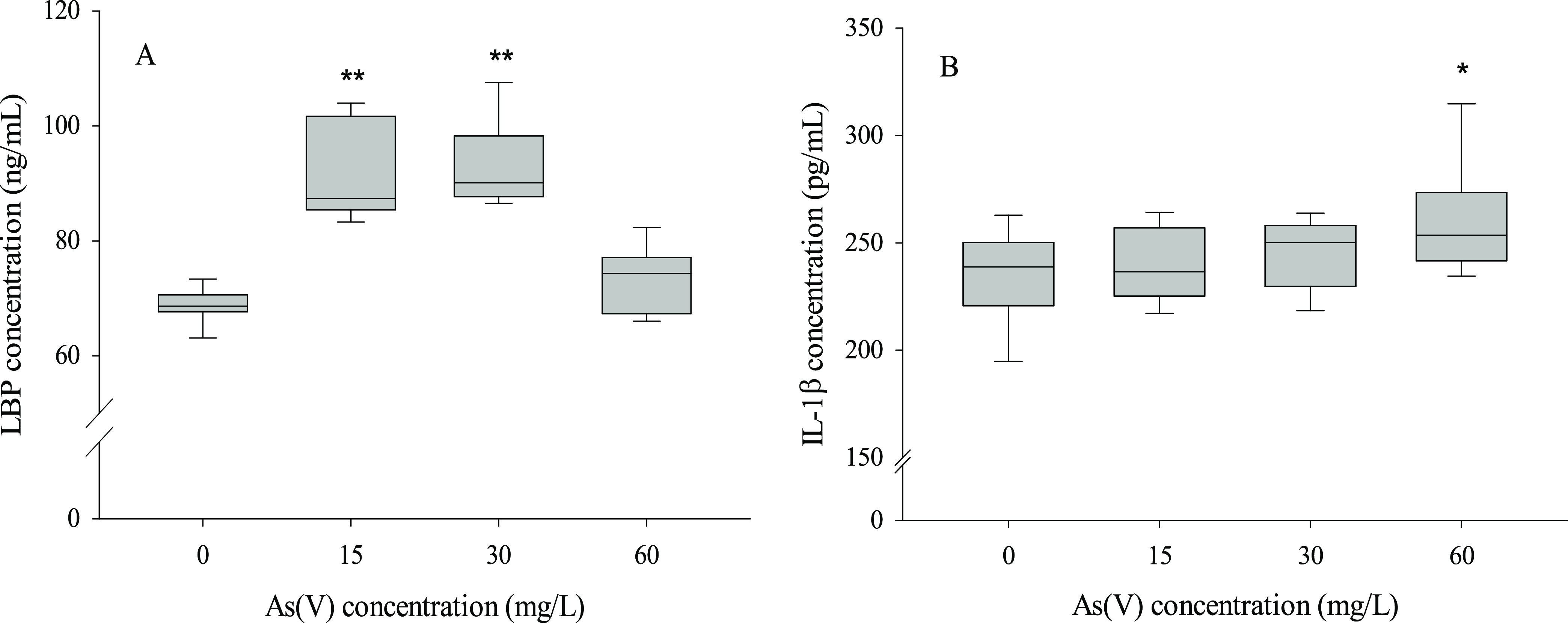
Serum markers of endotoxemia in the control and As(V)-treated animals. (A) LBP (ng/mL) and (B) IL-1β concentration (pg/mL). Values expressed as the mean ± SD (*n* = 9). Asterisks indicate statistically significant differences with respect to control animals (**, *p* < 0.001; *, 0.001 ≤ *p* < 0.05).

Endotoxemia was assessed by measuring LBP (Figure 10A) and proinflammatory cytokine IL-1β (Figure 10B) in serum. Exposure to arsenate increased serum LBP levels relative to control animals at 15 and 30 mg/L As(V) concentrations (35–36%). In addition, a statistically significant increase in serum IL-1β content was observed, although only at the highest concentration (10%).

### 3.7 Relationship between Several of the Toxicological Endpoints Assessed

**Figure 11:**
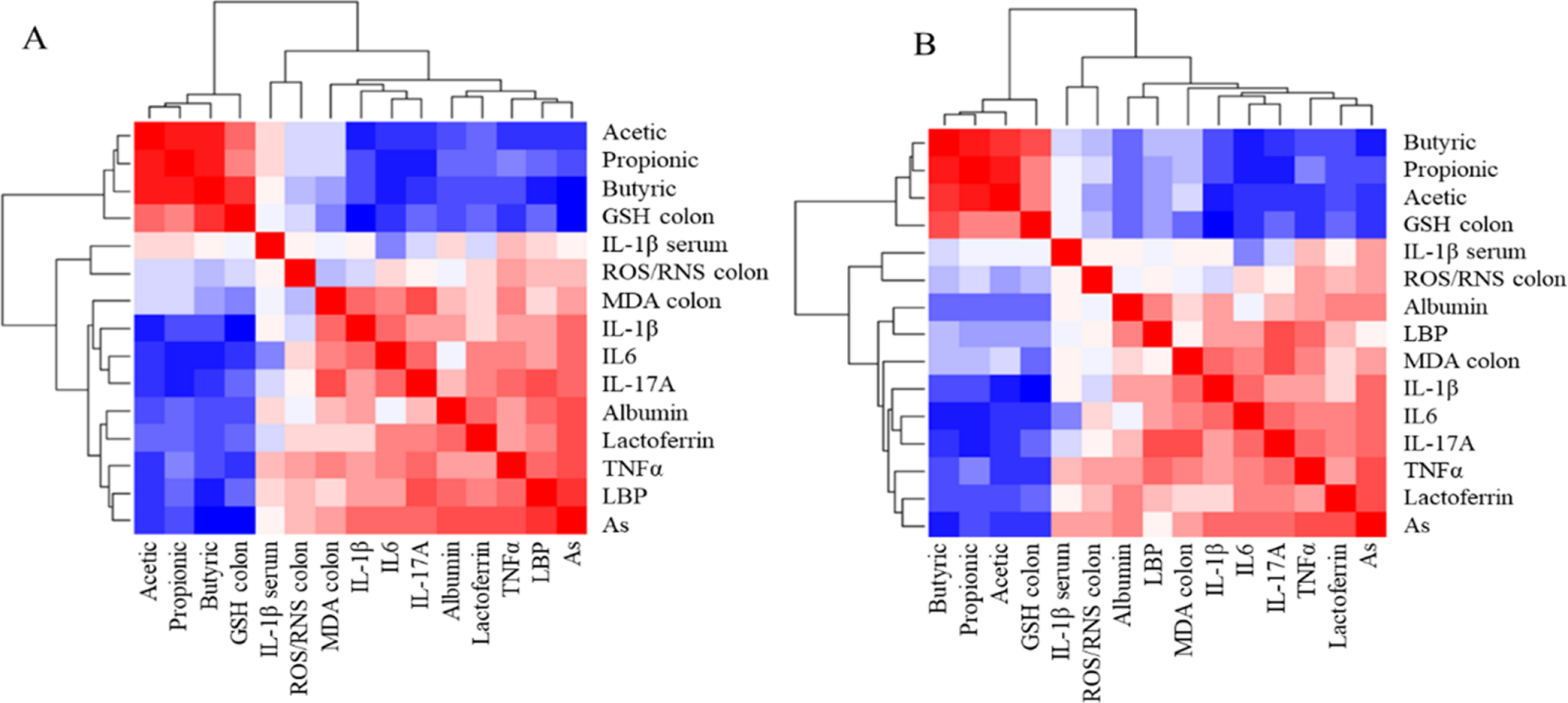
Heatmap representing the correlation matrix between several of the parameters analyzed in this study. (A) Map obtained by analyzing the control group and the animals treated with 15 and 30 mg/L As(V), (B) Map obtained by analyzing all groups. Red squares indicate a positive relationship between two variables, while blue squares indicate a negative relationship.

Figure 11 shows heatmaps of the correlation matrixes between some of the parameters analyzed. In graph Figure 11A, the correlation study was performed by removing the group of animals treated with 60 mg/L As(V); while in graph Figure 11B, the correlations were represented considering all groups. In general, a positive correlation between As(V) concentrations in drinking water and variables related to oxidative stress and intestinal inflammation was evidenced. These markers of toxicity, in turn, showed a negative correlation with luminal SCFA and colonic GSH contents, which correlated positively with each other. Intestinal permeability (fecal albumin) and the endotoxemia marker LBP showed an increase as As(V) concentrations and levels of some stress and inflammatory markers increased and as fecal GSH and SCFA levels decreased.

All of these correlations were more robust when the group of animals exposed to 60 mg/L was removed from the analysis (Figure 11A). It should be noted that some variables displayed a nonmonotonic dose–response (NMDR), i.e., the response to the metalloid follows an upward or downward trend, which is not linear in the animals treated with 60 mg/L As(V), which shows a reduced response.

## 4 Discussion

The present study shows that chronic exposure to As(V) affects the intestinal mucosa environment, altering its functionality. Animals treated with 30 and 60 mg/L As(V) through drinking water showed a significant increase in intestinal permeability compared with untreated animals (Figure 9). By contrast, although treatment with 15 mg/L As(V) resulted in increased oxidative stress (Figure 1A–C), some degree of leukocyte infiltration (Figure 3A,B) and downregulation of proteins of the junctional complex and the mucus layer (Table 2), it did not significantly affect intestinal permeability (Figure 9). Previous *in vitro* studies have shown that As(V) does not exert an effect on intestinal monolayers as relevant as As(III);^42^ however, in this study, exposure to As(V) generates a similar effect as As(III)^18^ in many of the toxicological endpoints analyzed. Transformations of As(V) as it passes through the digestive tract might partly explain the differences between *in vitro* and *in vivo* assays. Ingested As(V) may be reduced and/or methylated in the lumen due to the presence of reducing dietary substances,^16,43^ by the epithelial cells metabolism,^44^ or by the action of the gut microbiota.^45^ All of these abiotic or biotic factors transform As(V) into As(III) and other highly toxic methylated trivalent forms. These metabolites, which are more toxic than As(V), may ultimately cause damage to the intestinal environment. This could be the reason why As(V) toxicity is not so evident when assays are performed *in vitro*. Alternatively, the differences observed *in vitro* and *in vivo* may merely lie in the simplicity of the *in vitro* models, which comprise, in most cases, only absorptive epithelial cells of the intestine. This is opposed to the complex *in vivo* situation, where other cell types (including immune system cells) interact to provide a coordinated response to a toxic compound.

A differential toxic effect of both forms of inorganic As is observed at the gut microbiota level. Treatment of mice with As(III) affects the abundance of some taxa, although the composition of the microbiota is not altered.^18^ In the analysis of alpha diversity, differences in the effect of exposure to each of the arsenical forms were observed. Thus, while exposure to As(III) showed a tendency to increase diversity,^18^ exposure to As(V) had the opposite effect (Figure 5C,D). Also, the two arsenical forms had different effects on the composition of the microbiota (beta diversity). Exposure to As(V) caused significant changes in the composition that were not observed with As(III) exposure.^18^ Differential abundance analysis showed that the effects of As(V) were possibly genus- or strain-specific as no clear trends were evident at higher taxonomic levels.

Chronic exposure to As(V) also affects the fecal contents of SCFA, with a significant reduction in all three detected fatty acids (Figure 7A,B). All treatments with As(V) produce a similar reduction of these metabolites, with butyric acid being the most affected. The modification of the composition of the microbiota and the changes observed in some taxa could partly explain the alteration of the SCFA profile. In fact, *R. torques*, one of the groups showing reductions in As(V)-treated animals, is considered a major producer of butyrate^46^, and *Akkermansia*, also reduced by exposure to As(V), is an important producer of propionate.^47^ Additionally, the reduction in the luminal amount of SCFA may result from the effect of As(V) on the energy metabolism of the intestinal microbiota. Owing to the structural analogy between arsenate and phosphate, this inorganic form of As can disrupt several phosphate-dependent metabolic pathways.^48^ The formation and rapid hydrolysis of As(V)-ADP can lead to an uncoupling of oxidative phosphorylation, diminishing the ability of bacteria to produce ATP.^49^ In addition to an effect of As(V) on the microbiota composition or activity, the observed reduction in luminal SCFA levels might also be a consequence of increased uptake of these fatty acids by the intestinal epithelium. SCFAs are important energy molecules; indeed, butyrate is the primary energy source for colonocytes.^50^ Under stress conditions, such as those caused by chronic exposure to As(V), the energy demands of the intestinal cells may be increased, contributing to an increased level of butyrate consumption.

The effects of SCFA deficiencies were described previously. Two major signaling pathways linked to SCFAs have been characterized: protein-coupled receptors (GPRCs) and histone deacetylases (HDACs).^51^ The targeting of these pathways by SCFA promotes a series of events involved in homeostasis at the intestinal and systemic levels.^51^ SCFA plays an important role in the maintenance of the intestinal barrier. Previous studies have shown that butyrate regulates tight junctions through the modulation of the expression of tight-junction proteins^52^ and/or by the induction of their assembly.^53^ Therefore, the reduction of SCFA produced by As(V) exposure may be one of the causes of the increased permeability observed in the treated animals.

In addition to the microbiota and its metabolism, which could be considered the first and outermost component of the intestinal barrier, As(V) also affects other components that separate the intestinal lumen from the intestinal immune system. Gene expression data show a reduction in the number of transcripts of the major mucin of the colonic mucus layer. As observed in As(III)-treated animals,^18^ this reduction is associated with a reduction of the population of mucus-secreting cells. Exposure to As(V) could affect the differentiation of stem cells into the secretory lineage, as seen in As(III)-treated mice, which would explain the observed reduced number of mucus-secreting cells (Figure 8). Alternatively, it is also possible that As(V) exposure exerts a direct toxic effect on adult mucosecretory cells, affecting the synthesis of mucins essential for the maintenance of the intestinal mucus layer.

Regardless of the mechanism by which chronic As(V) treatment leads to lower *Muc2* expression, this toxic effect is an added difficulty for maintaining intestinal homeostasis and may be another mechanism of As(V) toxicity. It is interesting to note that reduced mucus production could affect so-called mucus-degrading bacteria. These components of the microbiota possess various enzymes that degrade mucus glycans for using these oligosaccharides as an energy source. These bacteria include, among others, *Akkermansia muciniphila*, *Bacteroides thetaiotaomicron*, *Bacteroides fragilis*, *Ruminococcus gnavus*, and *R. torques*.^54^ In the present study, we observed that some of these species have a lower relative abundance in As(V)-treated animals (Figures 6 and S3), which may be related to lower mucus abundance. In addition to this possible reduction in mucus contents, we observed reductions in the expression of intercellular junction proteins in animals chronically treated with As(V) (Table 2), which, as previously indicated, may be partly due to the luminal reduction of SCFA. These proteins are part of the molecular complexes that keep the epithelium sealed and limit paracellular transport.^55^ Therefore, we can conclude that the other major element (the epithelium and its secretome) that contributes to maintaining physical and chemical separation between the luminal contents and the intestinal immune system is also affected by As(V) exposure through drinking water.

The pattern of As(V) intestinal toxicity described here is similar to that described for As(III). Arsenate, or perhaps its metabolites, activates specific signaling pathways at the mucosal level that are related to inflammatory processes (p38, JNK, NFκB) (Figure 4). This activation may be a consequence of tissue damage, which generates a series of damage-associated molecular patterns (DAMPs) that activate and enhance this situation. The aforementioned kinase-dependent pathways are responsible for the synthesis of the proinflammatory cytokines that were found to be increased in tissues from As(V)-exposed animals (Figure 2). In addition to Th1 cytokines (IL-1β and TNF-α), it should be noted that a Th17 response was also found as As(V)-treated animals showed a significant increase in the cytokine IL-17A. This means that Th17 effector cells are induced in parallel to Th1 cells in chronic exposure to As(V).

Data on tissue contents of inflammatory cytokines suggest that inflammation is not necessarily continuous throughout the colon but that it can possibly be located at specific patches. This type of inflammatory patterning has been described in several inflammatory bowel disorders.^56^ The chronic proinflammatory and pro-oxidant process possibly tends to aggravate over time as the stimulus that originates it does not disappear, which could generate more DAMPs and proinflammatory molecules, some of which can also self-regulated, as is the case of TNF-α,^57^ which may exacerbate the magnitude of the process. Moreover, the increased permeability resulting from As(V) exposure makes the subepithelial immune system more exposed to substances that can activate it.

Finally, it should be noted that in As(V)-treated animals, an increase in some markers of endotoxemia is also observed (Figure 11A,B), suggesting the generation of a low inflammation steady state at the systemic level. This situation suggests that there may be some connections between intestinal disorders and the effects that this toxic element triggers at other locations.

## 5 Conclusions

This study has shown that exposure to As(V) through drinking water produces a toxic effect on the intestinal barrier similar to that of As(III),^18^ with a significant increase in permeability. However, this disruption is manifested at higher concentrations. As(V) mode of action may be linked to a pro-oxidant and proinflammatory response due to the activation of a number of signaling pathways by tissue damage molecular patterns, a phenomenon known as sterile inflammation.^58^ The only differential aspects of As(V) exposure with respect to As(III) are the changes in the composition of the microbiota. Effects on the bacterially produced SCFAs were also observed, which were slightly greater than those reported for As(III) exposure. This may be partly explained by the effect of As(V) on the abundance of certain intestinal microbial taxa. Considering that *in vitro* toxicity of As(V) in an intestinal epithelial cell model is less apparent, we could hypothesize that the manifestation of the *in vivo* toxicity is partly due to the transformation of the pentavalent inorganic form into more toxic species.

The results obtained in this study and in the previous study by Domene, Orozco, Rodríguez-Viso, Monedero, Zúñiga, Vélez, and Devesa^18^ show an effect of inorganic As in the intestinal tract, which could even influence the systemic toxicity, although further studies would be necessary to confirm this. The knowledge of the As mode of action at the intestinal level makes possible the implementation of strategies to reduce this damage, which may also impact As systemic toxicity.

## Supporting Information Available

Occult blood in feces of the control and As(V)-treated animals at weeks 19 and 22 (Table S1); test results for the homogeneity of multivariate dispersions and pairwise permutation tests using the betadisper and permutest functions in vegan (Figure S1); and relative abundance of selected genera in the different treatment groups, each dot represents an individual mouse fecal sample (Figure S2) (PDF)
