# Supplementary material for: Impact of Chronic Exposure to Arsenate through Drinking Water on the Intestinal Barrier
Source: Chem Res Toxicol. 2023 Oct 11;36(11):1731–44. doi: 10.1021/acs.chemrestox.3c00201 (PMC10726480; doi:10.1021/acs.chemrestox.3c00201)

SUPPORTING INFORMATION

Impact of a chronic exposure to arsenate through drinking water on the intestinal barrier

Adrián Domene, Helena Orozco, Pilar Rodríguez-Viso, Vicente Monedero, Manuel Zúñiga, Dinoraz Vélez, Vicenta Devesa*

Instituto de Agroquímica y Tecnología de Alimentos, Calle Agustín Escardino 7, 46980 - Paterna, Spain.

* To whom correspondence should be addressed (telephone (+34) 963 900 022; fax (+34) 963 636 301; e-mail: vdevesa@iata.csic.es)

Table of contents

Table S1. Occult blood in feces of control and As(V)-treated animals at weeks 19 and 22.......S2

Figure S1. Test results for homogeneity of multivariate dispersions and pairwise permutation tests using the betadisper and permutest functions in vegan..S3

Figure S2. Relative abundance of selected genera in the different treatment groups. Each dot represents an individual mouse fecal sample...S4

Table S1. Occult blood in feces of control and As(V)-treated animals at weeks 19 and 22

Treatment	Animal	Week 19	Week 22
Control	1	-	-
	2	-	-
	3	-	+
	4	-	-
	5	-	-
	6	-	-
	7	-	-
	8	-	-
	9	-	-
15 mg/L	1	-	-
	2	-	-
	3	-	+
	4	-	-
	5	+	+
	6	+	-
	7	+	+
	8	-	-
	9	+	+
30 mg/L	1	-	-
	2	+	+
	3	-	-
	4	-	+
	5	+	+
	6	-	+
	7	-	-
	8	+	+
	9	+	+
60 mg/L	1	+	+
	2	-	+
	3	+	-
	4	+	+
	5	+	+
	6	+	+
	7	-	+
	8	+	+
	9	+	-

Figure S1. Test results for homogeneity of multivariate dispersions and pairwise permutation tests using the betadisper and permutest functions in vegan.

```
Permutation test for homogeneity of multivariate dispersions
Permutation: free
Number of permutations: 999
Response: Distances
          Df   Sum Sq   Mean Sq      F N.Perm Pr(>F)
Groups     3 0.003217 0.0010723 0.6043    999  0.623
Residuals 32 0.056779 0.0017744
```

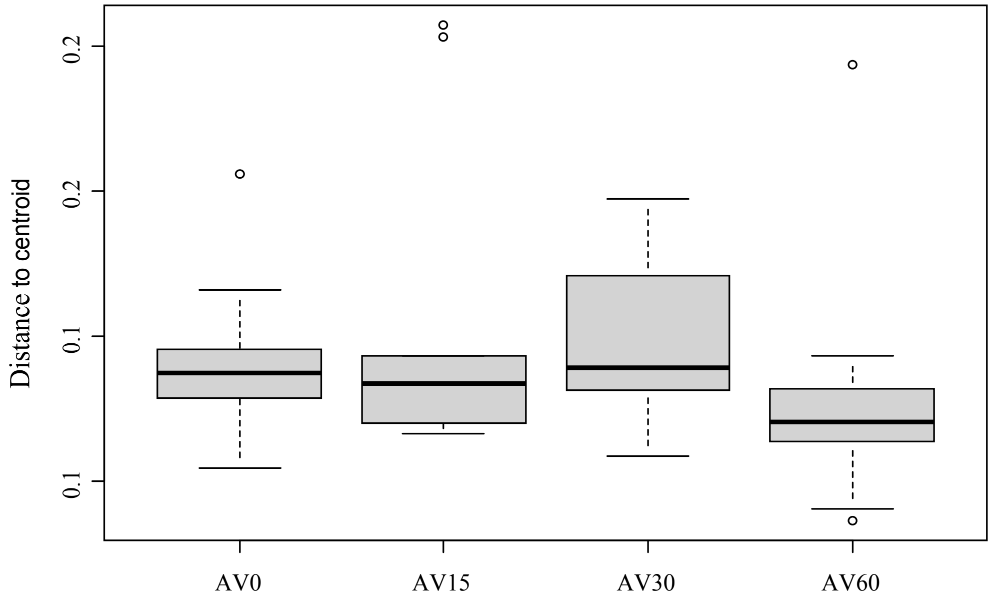

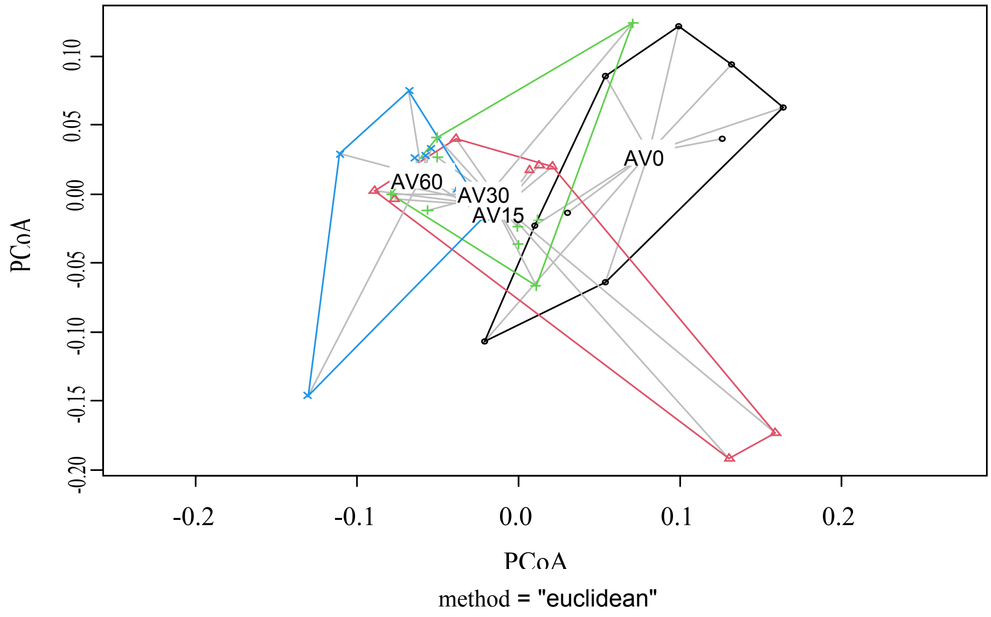

Figure S2. Relative abundance of selected genera in the different treatment groups. Each dot represents an individual mouse fecal sample

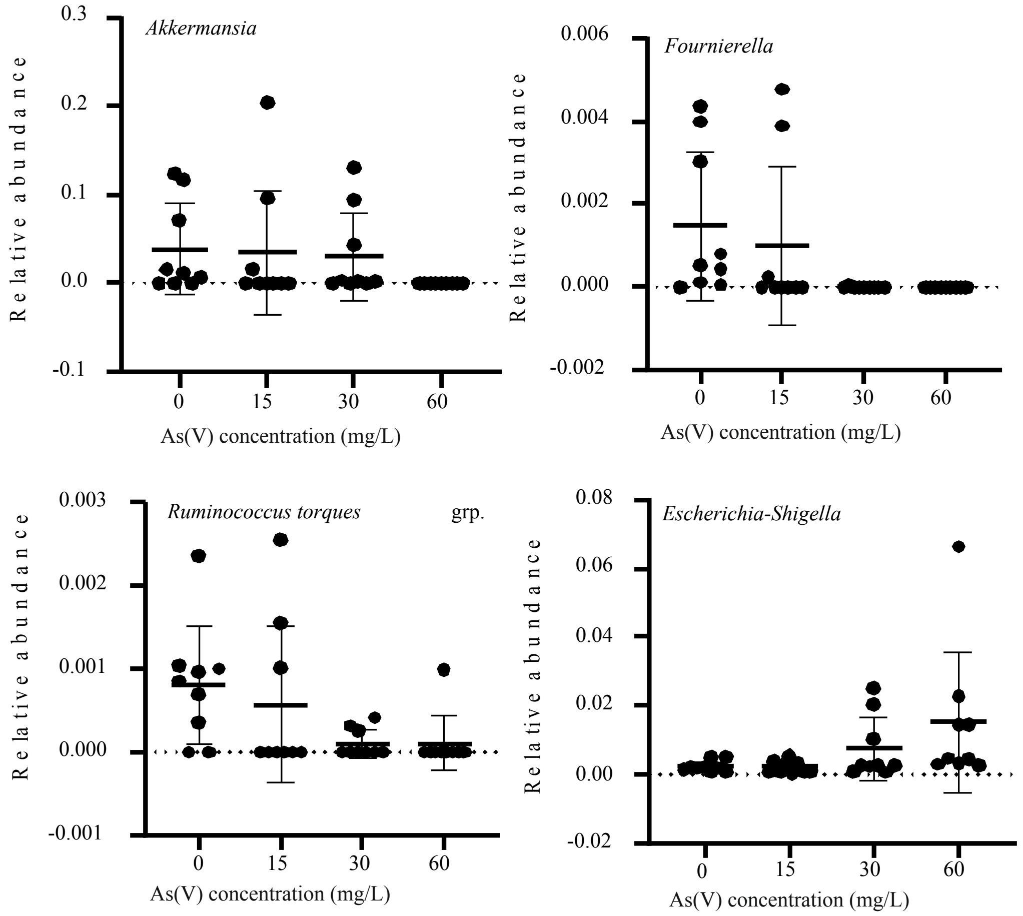

Supplement: Supplementary file 1 — tx3c00201_si_001.pdf [file tx3c00201_si_001.pdf]
